# Long-term surgical results of trabeculectomy for secondary glaucoma in Val30Met hereditary transthyretin amyloidosis

**DOI:** 10.1038/s41598-023-40029-4

**Published:** 2023-08-07

**Authors:** Junya Kitahara, Shinji Kakihara, Shuji Mukawa, Takao Hirano, Akira Imai, Teruyoshi Miyahara, Toshinori Murata

**Affiliations:** Department of Ophthalmology, Shinshu University School of Medicine, 3-1-1 Asahi, Matsumoto, Nagano 390-8621 Japan

**Keywords:** Medical research, Ocular hypertension

## Abstract

This study reports the long-term results of trabeculectomy (LEC) for secondary glaucoma in hereditary transthyretin (ATTRv) amyloidosis patients and its correlation with prior vitrectomy. A retrospective case series was conducted involving 31 consecutive eyes of 20 ATTRv amyloidosis patients who underwent LEC between 2007 and 2020. The mean follow-up period was 73.2 ± 37.0 months (range: 20–181 months). Postoperative intraocular pressures (IOPs) were evaluated based on the following criteria: (a) IOP between 6 and 21 mmHg without additional glaucoma surgeries, except for laser suture lysis, (b) IOP between 6 and 15 mmHg without additional glaucoma surgeries, except for laser suture lysis, and (c) IOP between 6 and 21 mmHg without additional glaucoma surgeries, except for needling and laser suture lysis. Kaplan–Meier analysis revealed survival rates after LEC of 0.52 at 36 months, 0.42 at 60 months, and 0.25 at 84 months under criterion (a); 0.49 at 36 months, 0.27 at 60 months, and 0.11 at 84 months under criterion (b); and 0.76 at 36 months, 0.71 at 60 months, and 0.65 at 84 months under criterion (c). Eyes with a history of small gauge transconjunctival vitrectomy (SGTV) exhibited a tendency towards lower survival rates, although no statistically significant difference was observed (log-rank test; p = 0.193 under criterion (a) and p = 0.0553 under criterion (b)). Our findings suggest that LEC and additional needling procedures can provide some control over IOP; however, the overall postoperative outcomes of LEC for ATTRv amyloidosis remain unsatisfactory, even in the era of SGTV with reduced conjunctival scarring.

## Introduction

Hereditary transthyretin (TTR) amyloidosis, also known as ATTRv amyloidosis, is a fatal neurodegenerative disease that usually affects adults and is caused by a mutated TTR variant^1^. Valine to methionine conversion at amino acid 30 (Val30Met) is the most common and well-studied among more than one hundred TTR variants^2^. Although previously believed to be confined to a few endemic areas, ATTRv amyloidosis has now been found worldwide. It is estimated that there are between 5,000 and 38,000 patients worldwide^3^.

Pupillary abnormalities, vitreous amyloidosis, and secondary glaucoma are known to develop on average 5.6, 9.5, and 14.5 years after systemic onset^4^. Although several effective systemic treatments improve life expectancy and quality of life^5,6^, they do not prevent the amyloid formation in the eye^7^. Therefore, ocular manifestations are becoming more common^7^.

Secondary glaucoma, which often develops after vitrectomy for vitreous amyloidosis and sometimes develops before vitrectomy, is reported to be difficult to treat^8–10^. According to reports, the results of trabeculectomy (LEC) are unsatisfactory due to the high rate of bleb encapsulation^11^. However, these reports do not reflect the recent approach of small gauge transconjunctival vitrectomy (SGTV), which does not require a large conjunctival incision and reduces conjunctival scarring.

We have been using SGTV for vitreous amyloidosis since 2005^12,13^. In this study, we report the long-term surgical results of LEC for secondary glaucoma in patients with ATTRv amyloidosis in the era of SGTV.

## Results

Table 1 summarizes demographic data, including age, gender, preoperative intraocular pressure (IOP), medication score, and visual field mean deviation, of the study cohort consisting of 31 eyes of 20 patients. The mean age of patients at the time of LEC was 51.0 ± 8.7 years. Of the patients, 18 underwent liver transplantation and one received tafamidis for systemic treatment, while 19 eyes out of 12 had a history of pars plana vitrectomy and 13 eyes out of 10 underwent phacoemulsification and intraocular lens insertion for cataracts. All eyes had open-angle glaucoma, and all surgeries were performed by specialized glaucoma surgeons (Te.M. and A.I.). The mean follow-up period after LEC was 73.2 ± 37.0 months. The mean preoperative IOP was 32.4 ± 8.8 mmHg, and the mean logMAR BCVA was 0.096 ± 0.52. The medication score was 4.5 ± 1.2, and the mean corneal endothelial cell density was 2478 ± 390 cells/mm^2^.

**Table 1:** Demographic and clinical characteristics of patients.

Parameter	Mean ± SD or No. (%)	95% CI or No. (%)
No. patients	20	
No. eyes	31	
Age, years	51.0 ± 8.7	47.8 to 54.2
Gender	Male, 12 (60)	Female, 8 (40)
BCVA,logMAR	0.096 ± 0.52	− 0.096 to 0.289
Preoperative IOP, mmHg	32.4 ± 8.8	29.2 to 35.7
Preoperative medication score	4.5 ± 1.2	3.99 to 4.91
Visual Field MD, dB	− 8.59 ± 8.67	− 11.77 to − 5.41
Corneal endothelium cell density, cells/mm^2^	2478 ± 390	2335 to 2620
Lens status	Phakia, 18 (58)	Pseudophakia, 13 (42)
Vitreous status	Vitreous, 12 (39)	Avitreous, 19 (61)
Surgical procedure	LEC, 30 (97)	LEC-PI, 1 (3)

*SD* standard deviation, *No* number, *BCVA* best corrected visual acuity, *logMAR* logarithm of the minimum angle of resolution, *IOP* intraocular pressure, *MD* mean deviation, *LEC* trabeculectomy, *PI* phacoemulsification and intraocular lens insertion.

**Figure 1:**
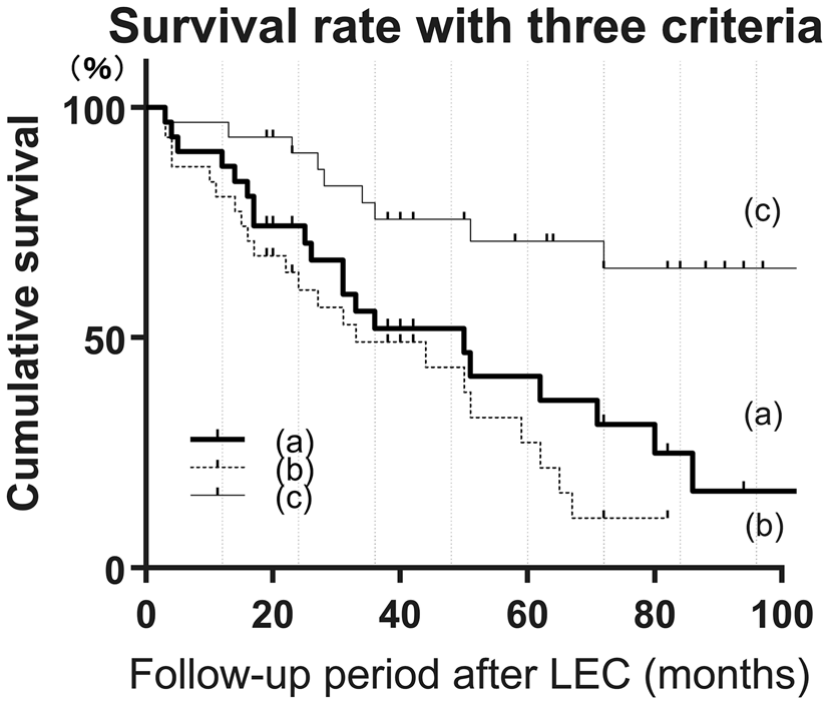
The Kaplan–Meier survival analysis demonstrates the overall survival rate after trabeculectomy (LEC). Surgical success was assessed based on postoperative IOPs as follows: (**a**) 6 ≤ IOP ≤ 21 and (**b**) 6 ≤ IOP ≤ 15, without additional glaucoma surgeries except for laser suture lysis, (**c**) 6 ≤ IOP ≤ 21 without additional glaucoma surgeries except for needling and laser suture lysis. Two consecutive deviations from the success criteria, loss of light perception, or additional glaucoma surgery were considered as failures in the Kaplan–Meier analysis. The overall survival rates after LEC were (**a**) 0.52 at 36 months, 0.42 at 60 months, and 0.25 at 84 months; (**b**) 0.49 at 36 months, 0.27 at 60 months, and 0.11 at 84 months; and (**c**) 0.76 at 36 months, 0.71 at 60 months, and 0.65 at 84 months after the operation, respectively.

The Kaplan–Meier survival analysis showed the overall survival rates after LEC to be (a) 0.52 at 36 months, 0.42 at 60 months, and 0.25 at 84 months; (b) 0.49 at 36 months, 0.27 at 60 months, and 0.11 at 84 months; and (c) 0.76 at 36 months, 0.71 at 60 months, and 0.65 at 84 months after the operation, respectively (Fig. 1, Supplemental Table 1).

**Figure 2:**
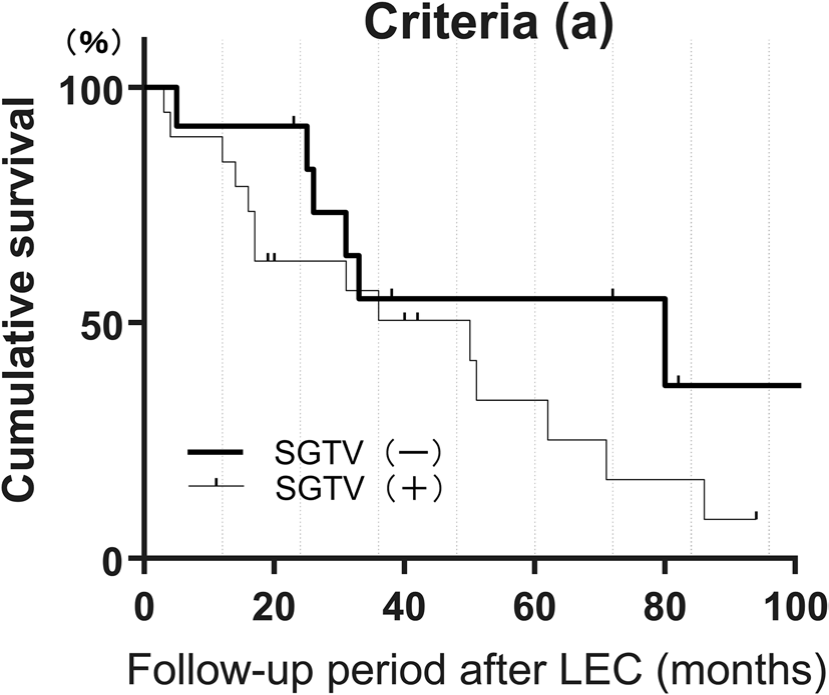
We compared the survival rate after LEC in eyes with and without previous SGTV, using criterion (a) to define surgical success as a postoperative IOP of 6 ≤ IOP ≤ 21 without additional glaucoma surgery other than laser suture lysis. For eyes without a history of SGTV, the survival rates after LEC were 0.55 at 36 months, 0.55 at 60 months, and 0.37 at 84 months after LEC. For eyes with a history of SGTV, the survival rates after LEC were 0.51 at 36 months, 0.34 at 60 months, and 0.17 at 84 months after LEC. More detailed data can be found in Supplemental Table 2.

In the assessment under criterion (a), survival rates after LEC for eyes without a history of SGTV were 0.55 at 36 months, 0.55 at 60 months, and 0.37 at 84 months. For eyes with a history of SGTV, the survival rates were 0.51 at 36 months, 0.34 at 60 months, and 0.17 at 84 months after LEC (Fig. 2, Supplemental Table 2). There was no significant difference in the Log-rank test (p = 0.193).

**Figure 3:**
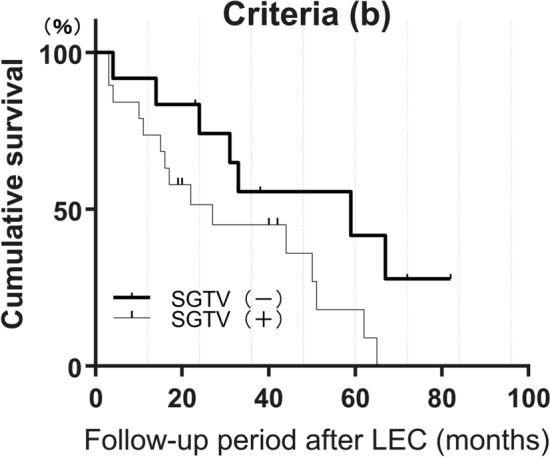
A comparison of the survival rates after LEC with and without previous SGTV, using criterion (b) was performed. Surgical success was defined as a postoperative IOP of 6 ≤ IOP ≤ 15 without additional glaucoma surgery other than laser suture lysis. The survival rates after LEC for eyes without a history of SGTV were 0.56 at 36 months, 0.42 at 60 months, and 0.28 at 84 months after LEC. The survival rates after LEC for eyes with a history of SGTV were 0.45 at 36 months, 0.18 at 60 months, and 0.00 at 84 months after LEC. More detailed data are provided in Supplemental Table 2.

In the assessment under criterion (b), the survival rates after LEC for eyes without a history of SGTV were 0.56 at 36 months, 0.42 at 60 months, and 0.28 at 84 months. For eyes with a history of SGTV, the survival rates were 0.45 at 36 months, 0.18 at 60 months, and 0.00 at 84 months after LEC (Fig. 3, Supplemental Table 2). There was no statistically significant difference between the two groups in the Log-rank test (p = 0.0553).

Postoperative interventions were summarized in Table 2. During the follow-up period, needling procedures were conducted in 17 eyes due to inadequate IOP reduction, while one eye underwent bleb revision. Additional glaucoma surgeries other than needling and bleb revision were conducted in nine eyes, including microhook ab interno trabeculotomy (3 eyes), LEC (2 eyes), Ahmed valve implantation (1 eye), Baerveldt glaucoma drainage implant surgery (2 eyes), and micropulse transscleral cyclophotocoagulation with Cyclo G6 (1 eye). SGTV was conducted in eight eyes.

**Table 2:** Postoperative Interventions in two groups.

Parameter	Previous SGTV (-), No. (%)	Previous SGTV ( +), No. (%)
No. eyes	12 (100)	19 (100)
Bleb revision	1 (8)	0 (0)
Needling	7 (58)	10 (53)
Glaucoma surgery	3 (25)	6 (32)
SGTV after LEC	6 (50)	2 (11)^#^

*SGTV* small gauge transconjunctival vitrectomy, *LEC* trabeculectomy.

^#^Additional SGTV for recurrent vitreous amyloidosis.

There was one eye that developed hyphema as a postoperative complication. Another eye had a shallow anterior chamber, but it did not require anterior chamber revision. During the follow-up period, the corneal endothelial cell density decreased by 30% or more in eight eyes compared to preoperative levels.

Following LEC and subsequent necessary IOP-lowering surgical interventions, the baseline IOPs of 32.4 ± 8.8 mmHg were significantly reduced to 12.4 ± 3.7 mmHg (31 eyes), 13.1 ± 3.5 mmHg (27 eyes), and 13.2 ± 3.2 mmHg (18 eyes) at 1, 3, and 5 years post-LEC surgery, respectively (all p-values < 0.0001 with Dunnett's test, as shown in Table 3).

**Table 3:** IOP and medication score time-course after trabeculectomy.

Time	IOP, mmHg	Medication score	Number of eyes
Baseline	32.4 ± 8.8	4.5 ± 1.2	31
1 year	12.4 ± 3.7	0.8 ± 1.4	31
3 years	13.1 ± 3.5	0.8 ± 1.3	27
5 years	13.2 ± 3.2	1.7 ± 1.4	18

Data are presented as mean ± standard deviation. Eyes with additional intervention for elevated IOP after trabeculectomy were not excluded.

*IOP* intraocular pressure.

Likewise, the medication score, initially 4.5 ± 1.2 at baseline, significantly decreased to 0.8 ± 1.4 (31 eyes), 0.8 ± 1.3 (27 eyes), and 1.7 ± 1.4 (18 eyes) at 1, 3, and 5 years after LEC, respectively (all p-values < 0.0001 with Dunnett's test, as presented in Table 3).

## Discussion

Recent advances in treatments for ATTRv amyloidosis have improved life expectancy and quality of life for patients^5,6^. However, the production of transthyretin in the eye can lead to ocular amyloidosis, which is a major concern^14^. Among various ocular manifestations, secondary glaucoma, and vitreous amyloidosis are the two main ocular issues^15^. Previous reports have shown an association between vitrectomy for vitreous amyloidosis and secondary glaucoma^9^. However, with the advancements in vitrectomy techniques and technology, it is important to re-evaluate the results of LEC in the current settings, considering the impact of conjunctival scarring on LEC^13,16,17^.

There are limited reports on postoperative results of glaucoma in ATTRv amyloidosis from a few centres. Long tube drainage surgeries such as Baerveldt glaucoma drainage implant surgery and Ahmed glaucoma valve implantation have relatively good long-term postoperative results^18–20^. Minimally invasive glaucoma surgeries targeting the trabecular meshwork can reduce IOPs after surgery and preserve the conjunctiva, but recent reports have shown that IOPs may increase about 2 years after surgery^21,22^. Filtration surgeries such as LEC and Ex-PRESS effectively reduce IOP, but their long-term effectiveness remains uncertain^11,23^.

In this report, we describe the results of LEC for secondary glaucoma in ATTRv in the SGTV era. The results suggest that LEC results for secondary glaucoma in ATTRv are generally poor, and SGTV may still impact the postoperative results of LEC, despite reduced conjunctival scarring. We believe that postoperative inflammation^24^ and dispersion of amyloid fibrils after vitrectomy^9^ may influence the results of filtration surgery, but we do not know the actual reasons for this. However, filtration surgery after vitrectomy seems to be considered a poor prognostic factor in general^25,26^. The long-term results of LEC were not satisfactory, resulting in the need for needling and loss of corneal endothelium in many cases. Although glaucoma drainage implant surgery has favourable results, we do not recommend it as the initial glaucoma surgery due to its inherent complications. Therefore, it is important to control secondary glaucoma in ATTRv amyloidosis with conventional glaucoma surgeries to delay glaucoma drainage implant surgery.

The present study has several limitations, including being a retrospective review of cases without a control group, varying follow-up periods after LEC, a small number of patients, and all patients having the TTR Val30Met variant. Additionally, both eyes of several ATTRv patients were included in the study. However, given the rarity of ATTRv amyloidosis, the results of this study are significant.

In summary, LEC and additional needling procedures can mildly control IOPs, but the overall postoperative results of LEC for ATTRv were not good, even in the era of SGTV with reduced conjunctival scarring^16^. It is essential to consider the pros and cons of each glaucoma surgery, such as LEC, Ex-PRESS, Ahmed valve, Baerveldt glaucoma drainage implant, and minimally invasive surgery, and choose the appropriate one for each patient^27,28^.

## Patients and methods

### Patients

We enrolled patients who were diagnosed with ATTRv amyloidosis (TTR Val30Met variant) and had received LEC treatment between 2007 and 2020. The Department of Medicine (Neurology and Rheumatology) at Shinshu University School of Medicine confirmed the diagnosis, as previously documented^29^. Before LEC treatment, a comprehensive ophthalmologic examination was performed, and eyes with a history of 20- or 23-gauge vitrectomy or surgery requiring conjunctival incision were excluded. Ultimately, our retrospective case series included 31 eyes from 20 patients. In eyes with a history of 25- or 27- gauge SGTV, vitrectomy procedures were performed as previously reported^13^.

### Trabeculectomy methods and follow-up

We used standard sub-Tenon anaesthesia with 2% lidocaine for all surgeries. To perform LEC, we made a limbal conjunctival peritomy in either the upper temporal or upper nasal quadrant and created a half-thickness 4 × 4 mm scleral flap. After the first scleral flap was made, we applied 0.04% mitomycin C for 4–5 min and rinsed it with a balanced salt solution. We then created a second flap inside the scleral bed of the first flap and removed the trabecular tissue en bloc with the second scleral flap.

After the iridectomy, we closed the first scleral flap with 4 to 7 sutures using 10–0 nylon. We reattached the conjunctiva using either 10–0 nylon or 7–0 virgin silk. For LEC alone, we induced miosis with topical 2% pilocarpine. If cataracts were present, we performed LEC after phacoemulsification and intraocular lens implantation through a clear corneal incision.

We examined all patients on postoperative days 1, 2, and 3, and then at intervals of 1–2 weeks for the first month, monthly for the next 6 months, and every 2 months thereafter. At each visit, we measured the IOPs using a Goldmann tonometer. To exclude the effects of postoperative IOP fluctuations, we excluded IOP measurements taken within 4 weeks of the LEC procedure.

### Outcome measures

Our first outcome measure is the cumulative survival rate after LEC. We categorized surgical success based on postoperative IOPs, as follows: (a) 6 ≤ IOP ≤ 21 without additional glaucoma surgeries except for laser suture lysis, (b) 6 ≤ IOP ≤ 15, without additional glaucoma surgeries except for laser suture lysis, and (c) 6 ≤ IOP ≤ 21 without additional glaucoma surgeries except for needling and laser suture lysis. In the Kaplan–Meier analysis, two consecutive deviations from the above success criteria, loss of light perception, or additional glaucoma surgery, were considered as death.

Our secondary outcomes are to investigate the association between a history of SGTV and postoperative results, as well as to identify postoperative complications. Additionally, we examined the changes in IOPs and medication scores in eyes affected by ATTRv amyloidosis that required LEC at different time points: baseline, 1 year, 3 years, and 5 years after LEC^30^. These alterations were evaluated in response to LEC and subsequent surgical interventions performed to reduce IOP. The calculation of medication scores followed the methodology described in the previously reported method^31^. It is important to note that eyes receiving additional interventions for elevated IOP following trabeculectomy were not excluded from the analysis.

### Demographic and clinical data of the patients

We obtained information from the medical charts regarding the patient's sex, age, lens status, best-corrected visual acuity (BCVA) using a Landolt ring chart, corneal endothelial cell density, IOPs, medication scores, results of visual field tests (using the Central 30-2 SITA Standard program with the Humphrey Visual Field Analyzer from Carl Zeiss Meditec), systemic treatment, and history of pars plana vitrectomy for vitreous amyloidosis. We converted BCVA to a logarithm of the Minimum Angle of Resolution (logMAR).

### Statistical analyses

We presented all continuous data as mean ± standard deviation with a 95% confidence interval. For statistical analysis, we used GraphPad Prism ver. 9.3.1 for Windows by GraphPad Software. We considered a p-value < 0.05 as statistically significant for all performed analyses.

### Ethics approval

Our study adhered to the principles of the Declaration of Helsinki and was approved by the Institutional Review Board of Shinshu University (Clinical research approval number: 4922). Written informed consent from each patient for publication was not required by the IRB approval. Instead, we posted the study protocol at the study institutions to notify participants about the study.

### Supplementary Information

Supplementary Table 1.

Supplementary Table 2.

## Data Availability

The datasets generated during and/or analysed during the current study are available from the corresponding author on reasonable request.

## Abbreviations

LEC: Trabeculectomy
SGTV: Small gauge transconjunctival vitrectomy
TTR: Transthyretin
ATTRv: Amyloidotic transthyretin variant
IOP: Intraocular pressure
