# Supplementary material for: Long-term surgical results of trabeculectomy for secondary glaucoma in Val30Met hereditary transthyretin amyloidosis
Source: Sci Rep. 2023 Aug 7;13:12755. doi: 10.1038/s41598-023-40029-4 (PMC10406936; doi:10.1038/s41598-023-40029-4)
Supplement: Supplementary file 1 — Supplementary Table 1. [file 41598_2023_40029_MOESM1_ESM.pdf]

Supplemental Table 1

Criteria (a)			Criteria (b)			Criteria (c)		
months	survival rate (%)	eyes at risk	months	survival rate (%)	eyes at risk	months	survival rate (%)	eyes at risk
0	100.0	31	0	100.0	31	0	100.0	31
3	96.8	31	3	93.5	31	3	96.8	31
4	93.5	30	4	87.1	29	13	93.5	30
5	90.3	29	10	83.9	27	19	93.5	29
12	87.1	28	11	80.6	26	20	93.5	28
14	83.9	27	14	77.4	25	23	90.1	27
16	80.6	26	15	74.2	24	27	86.5	25
17	74.2	25	16	71.0	23	28	82.9	24
19	74.2	23	17	67.7	22	34	79.3	23
20	74.2	22	19	67.7	21	36	75.7	22
23	74.2	21	20	67.7	20	38	75.7	21
25	70.5	20	22	64.2	19	40	75.7	19
26	66.8	19	23	64.2	18	42	75.7	18
31	59.4	18	24	60.4	17	50	75.7	17
33	55.6	16	27	56.6	16	51	70.9	16
36	51.9	15	31	52.9	15	58	70.9	15
38	51.9	14	33	49.1	14	63	70.9	14
40	51.9	12	38	49.1	13	64	70.9	13
42	51.9	11	40	49.1	11	72	65.0	12
50	46.7	10	42	49.1	10	82	65.0	10
51	41.5	9	44	43.6	9	84	65.0	9
62	36.4	8	50	38.2	8	88	65.0	8
71	31.2	7	51	32.7	7	91	65.0	7
72	31.2	6	59	27.3	6	94	65.0	6
80	24.9	5	62	21.8	5	97	65.0	5
82	24.9	4	65	16.4	4	103	65.0	4
86	16.6	3	67	10.9	3	127	65.0	2
94	16.6	2	72	10.9	2	136	65.0	1
115	0.0	1	82	10.9	1			
