# Supplementary material for: Long-term surgical results of trabeculectomy for secondary glaucoma in Val30Met hereditary transthyretin amyloidosis
Source: Sci Rep. 2023 Aug 7;13:12755. doi: 10.1038/s41598-023-40029-4 (PMC10406936; doi:10.1038/s41598-023-40029-4)
Supplement: Supplementary file 2 — Supplementary Table 2. [file 41598_2023_40029_MOESM2_ESM.pdf]

Supplemental Table 2

Criteria (a)				
	SGTV(-)		SGTV(+)	
months	survival rate (%)	eyes at risk	survival rate (%)	eyes at risk
0	100.0	12	100.0	19
3			94.7	19
4			89.5	18
5	91.7	12		
12			84.2	17
14			78.9	16
16			73.7	15
17			63.2	14
19			63.2	12
20			63.2	11
23	91.7	11		
25	82.5	10		
26	73.3	9		
31	64.2	8	56.8	10
33	55.0	7		
36			50.5	9
38	55.0	6		
40			50.5	8
42			50.5	7
50			42.1	6
51			33.7	5
62			25.3	4
71			16.8	3
72	55.0	4		
80	36.7	3		
82	36.7	2		
86			8.4	2
94			8.4	1
115	0.0	1		

Criteria (b)				
	SGTV(-)		SGTV(+)	
months	survival rate (%)	eyes at risk	survival rate (%)	eyes at risk
0	100.0	12	100.0	19
3			89.5	19
4	91.7	12	84.2	17
10			78.9	16
11			73.7	15
14	83.3	11		
15			68.4	14
16			63.2	13
17			57.9	12
19			57.9	11
20			57.9	10
22			51.5	9
23	83.3	10		
24	74.1	9		
27			45.0	8
31	64.8	8		
33	55.6	7		
38	55.6	6		
40			45.0	7
42			45.0	6
44			36.0	5
50			27.0	4
51			18.0	3
59	41.7	4		
62			9.0	2
65			0.0	1
67	27.8	3		
72	27.8	2		
82	27.8	1		
